# Supplementary material for: Family vulnerability scale: Evidence of content and internal structure validity
Source: PLoS One. 2023 Oct 25;18(10):e0280857. doi: 10.1371/journal.pone.0280857 (PMC10599550; doi:10.1371/journal.pone.0280857)
Supplement: S1 File — (DOCX) [file pone.0280857.s001.docx]

**S1 Supporting Information. Family Vulnerability Scale for Brazil (EVFAM-BR).**

| **Dimension** | **Item** | **Item score**  **1 (Yes) or 0 (No)** |
| --- | --- | --- |
| Income | 1. Is anyone in your household having financial difficulties? |  |
|  | 2. Is money short to meet household needs? |  |
|  | 3. Is it hard to secure access to different food types? |  |
| Healthcare | 4. Does anyone in your household use medication? |  |
|  | 5. Does anyone in your household use five types of medications, or more, daily? |  |
|  | 6. Does anyone in your household have a health condition that requires continuous care? |  |
|  | 7. Is anyone in your household impaired to perform daily activities? |  |
|  | 8. Does anyone in your household need help accomplishing daily healthcare procedures? |  |
| Family | 9. Did anyone in your household have an absent mother in childhood? |  |
|  | 10. Did anyone in your household have an absent father in childhood? |  |
|  | 11. Has anyone in your household been abandoned by the family? |  |
| Violence | 12. Does anyone in the household coexist with violent individuals? |  |
|  | 13. Has anyone in your household been the victim of violence? |  |
|  | 14. Is there any violence happening in your home? |  |
| **Total** | |  |
