# Supplementary material for: Family vulnerability scale: Evidence of content and internal structure validity
Source: PLoS One. 2023 Oct 25;18(10):e0280857. doi: 10.1371/journal.pone.0280857 (PMC10599550; doi:10.1371/journal.pone.0280857)
Supplement: S2 File — (DOCX) [file pone.0280857.s002.docx]

**S2 Supporting Information. Family Vulnerability Scale (*Escala de Vulnerabilidade Familiar*, EVFAM-BR, in Portuguese).**

| **Dimensão** | **Item** | **Escore Item**  **1 (Sim) ou 0 (Não)** |
| --- | --- | --- |
| Renda | 1. Alguém do domicílio passa por dificuldades financeiras? |  |
|  | 2. Falta dinheiro para atender as necessidades do domicílio? |  |
|  | 3. Existem dificuldades de acesso a diferentes tipos de alimentos? |  |
| Cuidado em Saúde | 4. Alguém no domicílio faz uso de medicamentos? |  |
|  | 5. Alguém no domicílio faz uso de 5 ou mais tipos de medicamentos por dia? |  |
|  | 6. Alguém no domicílio possui condição de saúde que requer cuidados contínuos? |  |
|  | 7. Alguém no domicílio tem dificuldades para realizar atividades do dia a dia? |  |
|  | 8. Alguém no domicílio necessita de ajuda para realizar seus cuidados diários de saúde? |  |
| Família | 9. Alguém no domicílio teve a mãe ausente durante a infância? |  |
|  | 10. Alguém no domicílio teve o pai ausente durante a infância? |  |
|  | 11. Algum familiar já esteve em situação de abandono pela família? |  |
| Violência | 12. Alguém no domicílio convive com pessoas violentas? |  |
|  | 13. Alguém em seu domicílio já foi vítima de violência? |  |
|  | 14. Acontece violência em sua casa? |  |
| **Total** | |  |
